# A computational model based on corticospinal functional MRI revealed asymmetrically organized motor corticospinal networks in humans

**DOI:** 10.1038/s42003-022-03615-2

**Published:** 2022-07-05

**Authors:** Eiji Takasawa, Mitsunari Abe, Hirotaka Chikuda, Takashi Hanakawa

**Affiliations:** 1 Department of Advanced Neuroimaging, Integrative Brain Imaging Center, National Center of Neurology and Psychiatry, Tokyo, Japan; 2 Department of Orthopaedic Surgery, Gunma University Graduate School of Medicine, Maebashi, Gunma Japan; 3 Department of Integrated Neuroanatomy & Neuroimaging, Kyoto University Graduate School of Medicine, Kyoto, Japan

**Keywords:** Network models, Spinal cord

## Abstract

Evolution of the direct, monosynaptic connection from the primary motor cortex to the spinal cord parallels acquisition of hand dexterity and lateralization of hand preference. In non-human mammals, the indirect, multi-synaptic connections between the bilateral primary motor cortices and the spinal cord also participates in controlling dexterous hand movement. However, it remains unknown how the direct and indirect corticospinal pathways work in concert to control unilateral hand movement with lateralized preference in humans. Here we demonstrated the asymmetric functional organization of the two corticospinal networks, by combining network modelling and simultaneous functional magnetic resonance imaging techniques of the brain and the spinal cord. Moreover, we also found that the degree of the involvement of the two corticospinal networks paralleled lateralization of hand preference. The present results pointed to the functionally lateralized motor nervous system that underlies the behavioral asymmetry of handedness in humans.

## Abstract (web summary)

MRI and network modelling reveal correlation between the degree of involvement of the two corticospinal networks and the lateralization of handedness in humans.

## Introduction

Hand dexterity is a remarkable ability characterizing higher primates, including humans who consistently use a preferred hand to perform manual dexterity tasks in daily activities^1–3^. For unknown reasons, the acquisition of hand dexterity parallels the lateralization of hand preference^1–3^. Hand movement is controlled mainly by the primary motor cortex (M1). M1 contralateral to the moving hand sends monosynaptic corticospinal connection to motoneurons in the spinal cord^4,5^. This direct corticospinal network develops in higher primates and plays a major role in the control of dexterous hand movement. M1s in bilateral hemispheres also connect to the multi-synaptic relay circuits in the brainstem and the upper cervical spinal cord, which send efferents to the spinal motoneurons controlling hands^4,5^. Although this indirect corticospinal network via the relay circuits had long been considered responsible only for gross forelimb movements in lower mammals, recent evidence suggests that they also participate in controlling dexterous hand movement in higher primates^4,5^. Comparative studies suggested that the replacement of the indirect corticospinal network by the direct corticospinal network likely paralleled the acquisition of hand dexterity^6^. The direct and indirect corticospinal networks are regarded as the phylogenetically new and old pathways, respectively^5,7^.

Recent studies implicated the inherently asymmetric organization of the corticospinal motor pathways underlying unilateral right-hand movement (RHM) and left-hand movement (LHM)^8,9^. It has been proposed that this network asymmetry may be associated with behavioral laterality, indicating handedness in daily motor activities^8,9^. However, it remains unclear how the two corticospinal networks work in concert to control hand movement. Moreover, it is unknown whether the organization of the two corticospinal networks relates to hand preference.

Here, we applied corticospinal functional magnetic resonance imaging (fMRI)^10–12^ to right-handed humans to estimate the recruitment of the two corticospinal networks during RHM or LHM. The present corticospinal fMRI techniques enable simultaneous measurements of activity in M1s and the spinal cord. By combining the corticospinal fMRI and computational modeling, we estimated corticospinal connectivity as a surrogate marker of the efferents from M1s onto the spinal cord segment at which the motoneurons innervating the hand muscles reside. We operationally constructed computational models consisting of the ‘direct’ corticospinal network that sends direct influences from the contralateral M1 onto the spinal cord and the ‘indirect’ network that converges influences from bilateral M1s. The ‘direct’ and ‘indirect’ corticospinal networks were conceptually in line with the concept of the ‘monosynaptic’ and ‘multi-synaptic’ corticospinal pathways, respectively, in non-human primates^5^. Both RHM and LHM primarily involve the functional connection between contralateral M1 and the spinal cord whereas LHM may recruit the functional connection between the ipsilateral M1 and the spinal cord more than RHM^13–15^. Together, we predicted the recruitment of the direct corticospinal network during both RHM and LHM and hypothesized a greater involvement of the indirect corticospinal network in LHM than in RHM in right-handed participants. We then investigated if interindividual differences in the involvement of the direct and indirect corticospinal networks paralleled the intersubject variability of the degree of right-handedness. Observations from a lesion study in non-human primates suggested that dexterous hand movements did not involve the direct corticospinal network once impaired non-preferred hand functions were recovered^16^. We therefore hypothesized that the non-preferred hand movement might involve the indirect corticospinal networks that originated from the ipsilateral M1. A behavioral laterality of the right-handedness was assessed by the questionnaire of Edinburgh handedness inventory (EHI)^17^.

## Results

### Asymmetry in activation patterns of the spinal cord and M1 during RHM and LHM

Thirteen young healthy adults performed a finger opposition task with RHM or LHM during the simultaneous measurement of blood oxygenation level-dependent (BOLD) signals from bilateral M1 and the spinal cord (see Supplementary Fig. 1). The finger opposition task that involved fractionated movements of separate fingers against the thumb. We employed finger opposition since it serves a building block of dexterous hand motor activities in our daily life^18,19^ and has been extensively used to examine different involvement of M1s in control of RHM and LHM^19–22^. First, we examined activity in M1s and the spinal cord at the level of C7-Th1 segments during RHM or LHM. Activity data were used to estimate network connectivity between M1 and the spinal cord in the following analyses. SHc activity was computed as averaged among BOLD signals in the right or left half of the spinal cord (SHc) at the level of the C5 or C7-Th1 segments. Motoneurons at C7-Th1 segments primarily innervate the hand muscles of interest. The C5 segment of the spinal cord was used as a control region to support the segment-level specificity of the measurement. We thus expected higher ipsilateral SHc activity at the C7-Th1 segments than at the C5 segment. We analyzed the effects of SEGMENT (C5 versus C7-Th1), SIDE (contralateral SHc versus ipsilateral SHc) and HAND (RHM versus LHM) on SHc activity using a three-way analysis of variance (ANOVA) (Supplementary Fig. 2). Multiple comparisons were performed with use of Bonferroni–Holm correction. These analyses replicated the findings of previous spinal fMRI studies^23–25^. The main effect of SEGMENT (i.e., C7-Th1 activity was greater than C5 activity; *F*_(1, 12)_ = 6.72; *p* = 0.024) and the interaction effect of SIDE * HAND (*F*_(1, 12)_ = 5.71; *p* = 0.034) were significant. We found such spinal segment-specific activity similarly for RHM and LHM. The SIDE-by-HAND interaction was significant only during RHM. In the analysis of the control C5 segment, the right and left SHc did not show increased activity during either RHM or LHM (*p* > 0.4 for all VOIs, see right part of the upper and lower panels, Supplementary Fig. 2). These findings agree with neurophysiological observations indicating that hand movements activate the motoneurons, interneurons, or other types of neurons mainly in the C6-Th1 segments^26^. However, note that broad spinal segments between C3 and Th1 are involved in hand movement in animal neurophysiological experiments^26^. It is possible that the current fMRI technique was not sensitive enough to detect small activity in the C5 segment, which could be shown with invasive electrophysiological techniques.

During RHM, the C7-Th1 segments showed higher activity in the right ipsilateral SHc (*p* = 0.013), but not in the left contralateral SHc (*p* = 0.44), compared to the C5 segment. Moreover, the C7–Th1 segment showed greater activity in the ipsilateral SHc than in the contralateral SHc (*p* = 0.008). During LHM, the C7-Th1 segments showed higher activity than the C5 segment in both the contralateral (*p* = 0.020) and the ipsilateral SHc (*p* = 0.040); comparable activity was observed between the ipsilateral and contralateral SHc (*p* = 0.72). These results confirmed segment-specific SHc activity during both RHM and LHM and lateralized SHc activity during RHM only. Bilateral SHc activity during LHM is consistent with previous reports that showed activation in both ipsilateral and contralateral SHc during LHM possibly because of the recruitment of interneurons of the contralateral SHc that contact on the motoneurons in the ipsilateral SHc^27,28^.

BOLD activity in M1 (Supplementary Fig. 3) also confirmed the findings from previous studies^29,30^. M1 activity was computed as averaged BOLD signals across activated voxels inside Brodmann area 4 (see Method). Both RHM and LHM mainly activated the contralateral M1 (family-wise error [FWE] corrected *p* < 0.05). Moreover, RHM deactivated ipsilateral M1 relative to the resting baseline (corrected *p* = 0.001 by small volume correction analysis) while a trend toward deactivation did not reach significance in the ipsilateral M1 during LHM (*p* > 0.1). The absolute value of the negative activity in the ipsilateral M1 was smaller during LHM than during RHM (paired *t* test, left M1 vs. right M1, *t*_12_ = −2.54, *p* = 0.026). This less pronounced deactivation of the ipsilateral M1 during LHM compared with during RHM was consistent with a previous study using a similar tapping task^30^. Consistently, many participants showed negative ipsilateral M1 activity during LHM although neither activation nor deactivation reached statistical significance at a group level because of interindividual variability (right panel in Supplementary Fig. 3). In summary, these results replicated the previous findings of independently measured M1 and SHc activities during unilateral hand movement^22–25,29,30^, endorsing the present simultaneous corticospinal fMRI methodology.

### Asymmetric recruitment of the network during LHM consisting of the spinal cord and M1, both of which are ipsilateral to the moving hand

Although both RHM and LHM primarily involve the contralateral M1-SHc network, previous brain stimulation studies suggested greater recruitment of the ipsilateral M1-SHc network during LHM than during RHM^13–15^. This apparent recruitment of the ipsilateral M1-SHc network occurs when a simple pattern of finger movements was repeated with the left hand^14^ and does not necessarily accompany ipsilateral M1 activity above the resting baseline (Supplementary Fig. 3)^30^. Indeed, we did not observe ipsilateral M1 activity during LHM at a group level, but this does not mean that the ipsilateral M1-SHc network was not involved in LHM. We conjectured that the functionality of the ipsilateral M1-SHc network connectivity should exert influence on the ipsilateral SHc activity even without ipsilateral M1 activation or deactivation at a group level.

**Fig. 1:**
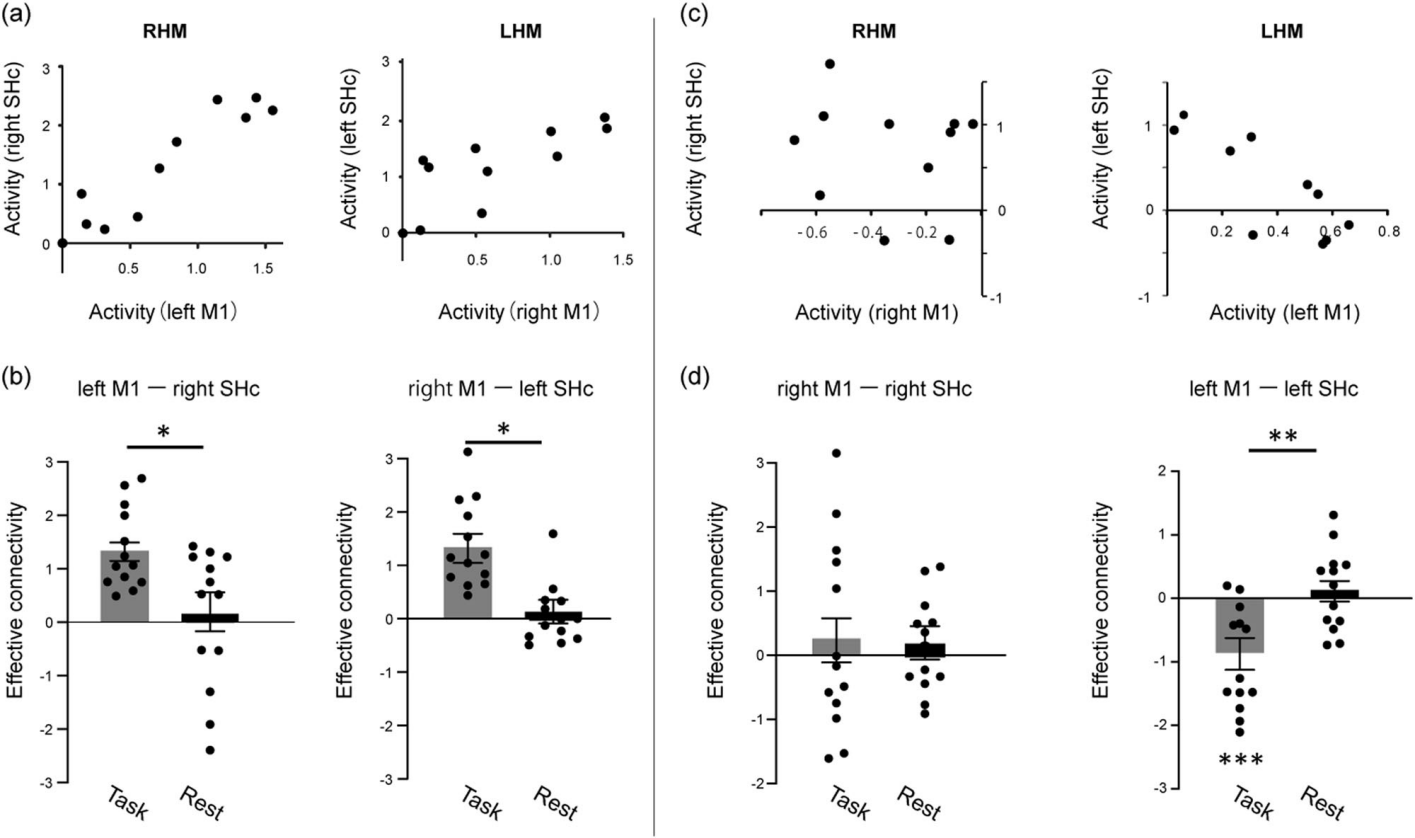
Asymmetric recruitment of the ipsilateral corticospinal network during hand movement. **a** Correlation between activity in contralateral M1 (*x*-axis) and ipsilateral SHc (*y*-axis) during RHM (left panel) or LHM (right panel) in a representative participant. Dots indicate the mean activity at each time point during hand movements. **b** Effective connectivity (contralateral M1–ipsilateral SHc) for RHM and LHM during the task and during rest periods. *p < 0.001. **c** Correlation between ipsilateral M1 activity (*x*-axis) and ipsilateral SHc activity (*y*-axis) in the same representative participant. **d** Effective connectivity (ipsilateral M1–ipsilateral SHc) for RHM and LHM during the task and rest periods (**p = 0.03, *** indicates *p* = 0.01).

We analyzed the task-induced modulation of effective connectivity between the M1 and the ipsilateral SHc. A simple linear regression analysis was performed on individual’s data, assigning M1 activity (Supplementary Fig. 3) to the explanatory variable and the ipsilateral SHc activity at the level of C7-Th1 segments (Supplementary Fig. 2) to the independent variable (Fig. 1a). The regression slope was taken as a measure of effective connectivity, which provides an estimation of an input-output function^31^. Changes in effective connectivity were compared between RHM or LHM and at rest, using a Wilcoxon signed-rank test. Functional connectivity was also computed to confirm the statistical correlation of activity (i.e., correlation coefficient) between M1 and ipsilateral SHc during RHM or LHM relative to rest (Supplementary Figs. 4, 5 and supplementary note 1). The effective connectivity of the contralateral M1-SHc network was positive during both RHM and LHM (Fig. 1b and Supplementary Fig. 4), supporting that both RHM and LHM involve the contralateral M1-SHc network. The effective connectivity of the ipsilateral M1-SHc network did not differ between RHM and rest (Fig. 1c, d and Supplementary Fig. 5). Contrarily, the effective connectivity of the ipsilateral M1-SHc network was significantly negative during LHM than the rest, demonstrating an inverse relationship between the ipsilateral M1 activity and the ipsilateral SHc activity (Fig. 1c, d and Supplementary Fig. 5). Consistently, transcranial magnetic pulses over the ipsilateral M1 at a resting state suppress the ipsilateral SHc motoneurons, suggesting that the ipsilateral M1-SHc network exerts overall inhibitory influences on the motoneurons^32^. These findings support the involvement of the ipsilateral M1-SHc network during LHM^13–15^ even without group-level activation or deactivation of the ipsilateral M1 (Supplementary Fig. 3). This influence is probably mediated by the combination of excitatory and inhibitory effects underpinned by the reticulospinal, propriospinal, and segmental interneuronal tracts^4^. We interpreted that the ipsilateral M1-SHc network was involved in the LHM and mediated the inverse correlation between ipsilateral M1 activity and ipsilateral SHc activity (Fig. 1). Evidence from non-human primates indicated that signals from contralateral M1 modulated activation of the relay neurons^33^ that may modulate transsynaptic efficacy of the ipsilateral M1-SHc network^4^. We then tested the dependency of the ipsilateral M1-SHc effective connectivity on the contralateral M1 activity. This analysis indicated that a higher contralateral M1 activity was coupled with a more negative value of the effective connectivity of the ipsilateral M1-SHc network during LHM (*r* = −0.58, *p* = 0.03; Supplementary Fig. 6). This relationship was not clear during RHM, however (*p* = 0.39). Our results suggested the neural circuit that sends signals from the contralateral M1 to the ipsilateral M1-SHc network. Previously, it was proposed that interhemispheric influences between M1s might modulate the recruitment of the ipsilateral M1-SHc network^34,35^. It is hence possible that interhemispheric connectivity between the bilateral M1s could influence the corticospinal connectivity differently between RHM and LHM here. We tested this possibility but did not find any task-induced modulation of interhemispheric effective connectivity during either RHM or LHM (*p* > 0.3 for both hands; Supplementary Note 1). This finding is consistent with recent reports that, although gross hand movements induce an increased effective connectivity between bilateral M1s^21,36^, fine hand movements do not^37^. Therefore, it may be surmised that interhemispheric effective connectivity between bilateral M1s was less likely to be involved in the modulation of M1-SHc networks in the finger-tapping task tested here.

### Involvement of the indirect corticospinal network pathway integrating the contralateral and ipsilateral M1-SHc networks in LHM

**Fig. 2:**
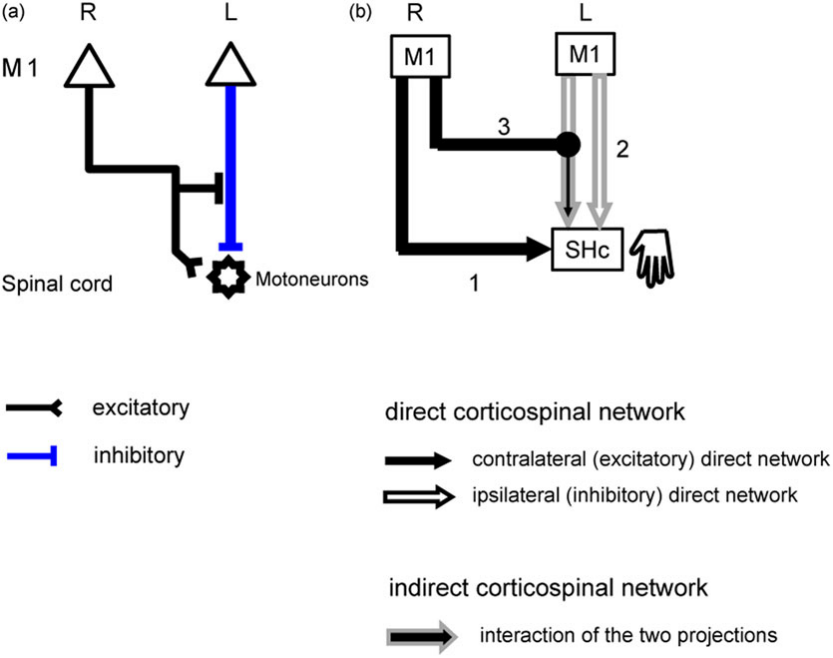
Effective connectivity between M1 and SHc and a proposed network model for LHM. **a** The positive effective connectivity underpins the net excitatory influences onto the motoneurons (black line; see also Fig. 1b), and negative effective connectivity represents the net inhibitory effects on motoneurons (blue vertical line; see also Fig. 1d). Signals from right contralateral M1 may exert modulation of the negative effective connectivity (black horizontal line, see also Supplementary Fig. 6)^33^. Neurophysiological evidence suggested interaction between effective connectivity of the contralateral M1−SHc and the ipsilateral M1−SHc networks that occurred at the brainstem or the spinal cord^32^. **b** The direct corticospinal network models were constructed with the single influences from right M1 (black line) or left M1 (white line with gray outline) onto SHc. The indirect corticospinal network model was designed to integrate the influences from left and right M1 onto SHc (black line with gray outline).

Our findings indicate that LHM involved more complicated networks consisting of both the contralateral M1-SHc and the ipsilateral M1-SHc networks than did RHM. Anatomical evidence supports the indirect corticospinal network connecting between these two M1-SHc networks^4^. We thus used computational modeling to test the assumption that information from both M1s would be integrated to modulate ipsilateral SHc activity during LHM (Fig. 2). Two possible forms of interactions between the contralateral M1-SHc and the ipsilateral M1-SHc networks influencing SHc activity were tested separately according to network model theories: an interaction of “activity” between bilateral M1 or an interaction of the “connectivity” of the two M1 networks. First, we tested the activity model in which the indirect corticospinal network might integrate the effects of M1 activity onto ipsilateral SHc. However, we were not able to identify any activity models that explained SHc activity (*p* > 0.2 for all models, see Supplementary Fig. 7).

**Fig. 3:**
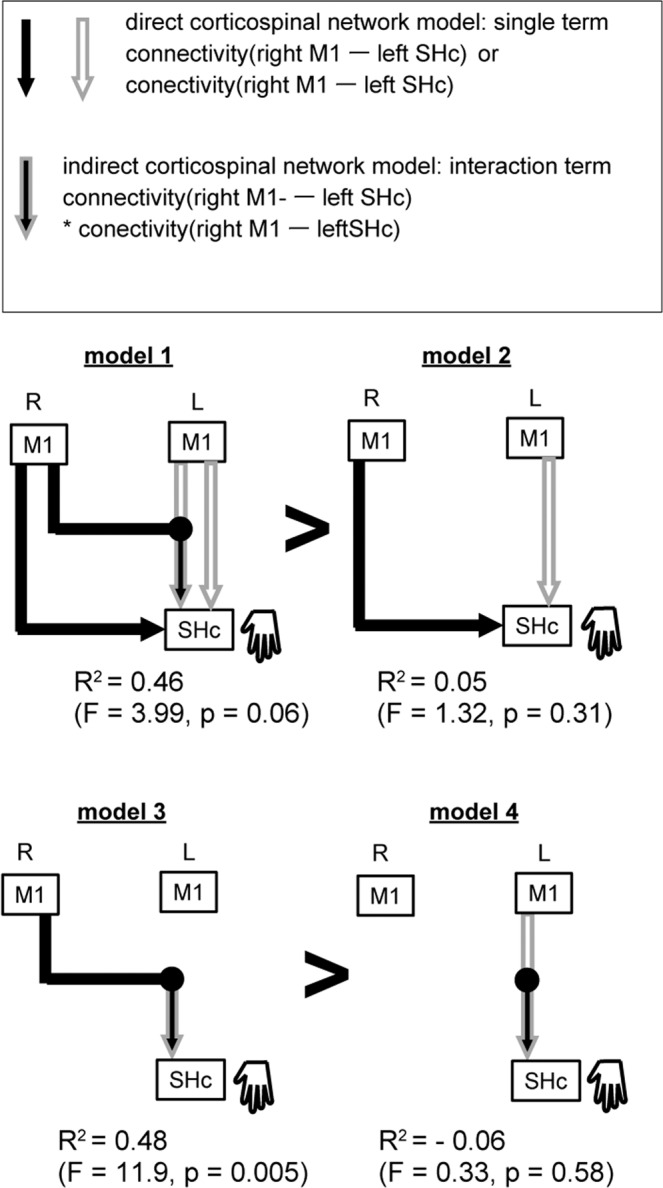
Network models of SHc activity during LHM with single or interaction terms. Single terms (black line and white line with gray outline) and interaction terms (black line with gray outline) in the connectivity model. According to structural equation modeling, single and interaction terms modulated by contralateral M1 activity or ipsilateral M1 activity were used to model the direct and indirect corticospinal networks. Model 1 is the full model that contains the two interaction terms and model 2 only contains the two single terms. See Supplementary Fig. 8 for model equations. The *R*^2^ values were adjusted (see “Methods”).

Next, we tested the connectivity model. According to the computational model referred to as the structural equation modeling, the net connectivity of the indirect corticospinal network was modeled as the interaction of the connectivity of the contralateral and ipsilateral M1-SHc networks^38,39^. The interaction term represents the mathematical multiplication of the connectivity of the two networks. Previous reports suggested the different roles of the contralateral M1 and the ipsilateral M1 for controlling hand movements^13–15^. The interaction terms were thus designed to be modulated by either the contralateral M1 activity or the ipsilateral M1 activity. Our results (Fig. 1) revealed that LHM involved both the contralateral M1-SHc and the ipsilateral M1-SHc networks. We thus constructed the single terms modeling each M1-SHc network. We tested whether the full model with the indirect corticospinal network might explain SHc activity during LHM more than the model consisting of the direct corticospinal networks only. We compared the fitting of the network models (Fig. 3), using adjusted R^2^ values along with Akaike information criterion and Bayesian information criterion (see “Methods”). The full network model with the two interaction terms (Fig. 3, model 1; Supplementary Fig. 8, equation *a*) was marginally significant (*R*^2^ = 0.477, *p* = 0.055), and seemed to explain SHc activity better than the model with both direct corticospinal networks only (*R*^2^ = 0.06, *p* = 0.31) (model 2, equation *b*). The comparison between model 1 and model 2 with the use of the bootstrap procedure indicates that the interaction terms corresponding to the indirect corticospinal network improved the explanation of SHc activity (see the comparison labeled “δ“ in Supplementary Fig. 8). Anatomical evidence suggests that the multi-synaptic pathways between the M1s and the spinal cord are the substrates of the indirect corticospinal network^4^. Transcranial magnetic stimulation applied over both M1s increases activity in the spinal motoneurons although this increase was smaller than the stimulation applied over the contralateral M1 only^32^. This report indicated that inputs from bilateral M1s induced a net increase of activity in the spinal cord. Our results unveiled that the indirect corticospinal network likely mediated the increase of SHc activity even though the ipsilateral M1 showed a trend toward deactivation (Supplementary Fig. 3). The indirect corticospinal network might intervene the inverse relationship between activity in the ipsilateral M1 and the spinal cord observed during LHM (Supplementary Figs. 1, 3).

### Individual differences in the involvement of the direct and indirect corticospinal networks paralleling intersubject variability in the lateralization of hand preference

**Fig. 4:**
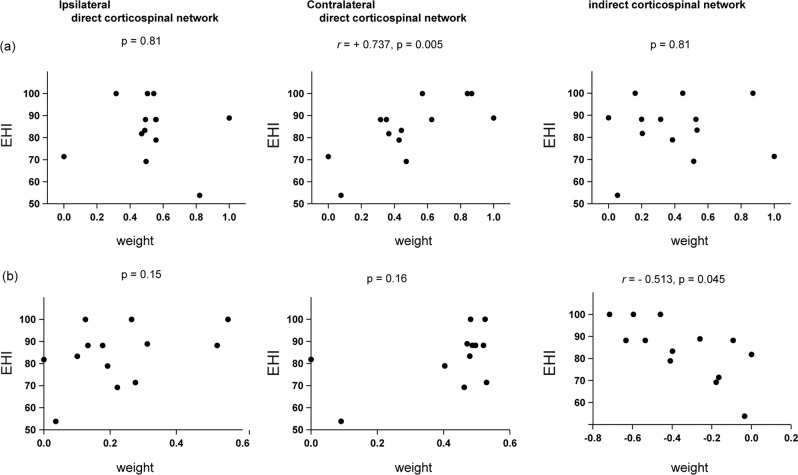
Correlation between the involvement of the direct or indirect corticospinal networks in hand movement and individual variability in the degree of hand preference. For each individual, we computed the weights for each of the single terms and interaction terms. The scatter plots represented the relationship between the weight for each component (*x*-axis) and the EHI score (*y*-axis). **a** The model for RHM. **b** The model for LHM.

No previous studies examined the degree of involvement of the direct or the indirect corticospinal networks in the preferred hand or the non-preferred hand movement in humans. Observation from a lesion study suggested that the preferred hand movement primarily involved the contralateral frontal cortices when the laterality of hand preference was higher. However, this was not necessarily the case when the laterality of hand-use was ambiguous (i.e., almost ambidextrous)^16^. This observation hinted that hand movement might recruit frontal cortices in the ipsilateral hemisphere when the moving hand was not usually used for dexterous hand motor tasks. We expected greater involvement of the direct corticospinal network in the preferred hand movement than in the non-preferred hand movement. We also tested that the involvement of the indirect corticospinal network may differ, depending on the hand used regularly for daily dexterous motor activities (preferred hand) or not (non-preferred hand). Given that this was the case, the interindividual variability in the involvement of the direct and indirect corticospinal networks may be correlated with that of the degree of preference for each hand. To test this, we first performed an individual-level simulation of the involvement of each corticospinal network during each hand movement. We constructed the full model that included the two direct corticospinal networks and the indirect corticospinal network (Fig. 2). We used the same model for RHM and LHM (Fig. 4), but the weight for each component, indicating the degree to which each component might explain SHc activity, was optimized for each participant (Supplementary Note 2). Our results indicated that the contralateral direct corticospinal network was recruited to a greater degree during RHM than during LHM (Supplementary Fig. 9). These data point to the functionally lateralized organization of the direct and indirect corticospinal motor networks for RHM and LHM, which we thereby further assessed. The degree of preference for the right hand was estimated using the Edinburgh Handedness Inventory (EHI) questionnaire. A higher EHI score indicates a strong preference for the use of the right hand as well as the non-use of the left hand in daily dexterous hand motor activities^17^. During RHM, a higher EHI score was correlated with a greater weight for the term modeling the direct contralateral corticospinal network (*r* = +0.737, *p* = 0.005; Fig. 4a). The other weights were not correlated with the EHI score during RHM. We were aware that the weight on the indirect corticospinal network term was similar to that on the direct pathway term during RHM (middle and most-right panel, Fig. 4a). Our results did not exclude the possibility that RHM might recruit the indirect corticospinal network in some individuals, although the group-level analysis did not support the role of the indirect pathway in the RHM (Fig. 1). This could be ascribed to interindividual variability in the recruitment of the indirect pathway in RHM. During LHM, a higher EHI score was correlated with a greater weight on the interaction term corresponding to the indirect corticospinal network (*r* = −0.5139, *p* = 0.045; Fig. 4b). We have acknowledged the negative weight on the indirect corticospinal network in this analysis. The net connectivity of the indirect corticospinal network (i.e. interaction {[connectivity(contralateral M1–ipsilateral SHc) * connectivity(ipsilateral M1–ipsilateral SHc)] }) showed negative (Supplementary Fig. 8). The influences from bilateral M1s onto the spinal cord are interpreted net positive in the indirect corticospinal network model since this computation came from the results of the mathematical multiplication between the negative sign of the weight and the negative sign of effective connectivity. The other weights were not correlated with the EHI score during LHM. These results indicate that the involvement of the direct corticospinal network in RHM is correlated with an individual’s preference for the right hand. Notably, the involvement of the indirect corticospinal network in non-preferred LHM paralleled the lateralization of hand preference. Lastly, we confirmed that the interhemispheric connectivity between bilateral M1, or bilateral M1 activity itself, was not associated with hand preference (Supplementary Note 3).

## Discussion

Recent advances in MRI techniques have enabled the simultaneous measurement of activity in the brain and the spinal cord, facilitating research that investigates functional interactions underlying the top-down motor control or sensory processing^10–12,40^. However, these studies did not address the relationship between the organization of the brain-spinal networks and the behavioral phenotypes uniquely in humans, such as hand preference. We first demonstrated asymmetric recruitment of the corticospinal connectivity between RHM and LHM. Using the network modeling, we were able to estimate recruitment of the network components corresponding to the direct corticospinal networks that sent influences on the spinal motoneurons from the M1 as well as the indirect corticospinal network that integrated influences from bilateral M1s. Finally, we were able to link these findings to individual hand preferences in daily activities. Our results have provided insights into the organization of the lateralized motor nervous system underlying the asymmetry of motor daily activities in humans.

Uniquely among primates, humans exhibit strongly lateralized motor corticospinal pathways^41^, although no established theory has yet explained the biological mechanisms underlying this lateralization. Nevertheless, recent studies have provided hints of the asymmetric organization of the human motor nervous system even at the prenatal stages. For example, fetuses already exhibit lateralized arm movements at 10 weeks’ gestation^2^, despite fiber tracts connecting between the forebrain and the spinal cord have not been established yet at this stage^42^. Gene expression profiles have indicated that the maturation of the spinal cord occurs at earlier stages of postconception than that of the forebrain^8,9^. Moreover, maturation patterns of the neural architectures differ between the right and left SHc; namely, the left SHc matures earlier than does right SHc. Further evidence has implicated the maturation of the multi-synaptic corticospinal connections at the prenatal term^43^ and this maturation probably occurs earlier than the establishment of the monosynaptic corticomotoneuronal connection^44^. It is thus possible that early-maturing left SHc motoneurons may receive stronger innervation from the indirect corticospinal network than from the direct corticospinal network^8,9^. This idea follows the theory that “ontogeny recapitulates phylogeny”^41,45^. In contrast, staggered maturation of the right SHc would make it suitable for the direct corticospinal tract from the left M1 to innervate motoneurons^44^. This predominant innervation from the contralateral corticospinal tract might underlie the greater involvement of the contralateral direct pathway in controlling RHM rather than LHM (Supplementary Fig. 9). It is possible that the genetically programmed staggered maturation of the spinal cord triggers the asymmetrical organization of the network architectures between M1 and SHc for controlling unilateral hand movement. This “spinal cord first” theory of motor laterality signifies the necessity to examine spinal cord activity simultaneously with M1 activity in the study of motor laterality. Starting from neonatal stages, furthermore, use-dependent changes can also be observed in the corticospinal projections between M1s and the spinal cord^42,46^. To understand the neurobiological machinery underlying handedness, it is thus important to investigate corticospinal activity and connectivity altogether in humans longitudinally in the future. The present study developed a non-invasive methodology to pave such a research venue toward this proposal.

We proposed a neurocomputational model of the direct and indirect corticospinal networks that were asymmetrically organized for RHM and LHM in right-handed humans. Indeed, neurophysiological and imaging studies partly supported our results in right-handed healthy participants^13–15^, indicating that the contralateral M1 serves the execution of both RHM and LHM. The ipsilateral M1 participated especially in LHM^13,14^ although involvement of the ipsilateral M1 varies with tasks^30,47,48^. Integration of signals from bilateral M1s was observed during LHM but not RHM, and likely occurred at the brainstem or the spinal cord^32^. These lines of evidence corroborated the functionally lateralized direct and indirect corticospinal networks in right-handed humans. The models of the direct and indirect corticospinal networks came from the evidence of the monosynaptic or multi-synaptic corticospinal pathways in non-human primates^5^. Using the corticospinal fMRI techniques, we applied the network models to human data. The present results supported that the direct and indirect corticospinal networks are likely implemented for controlling hand movements commonly across primates. The two pathways may be referred to as the phylogenetically new and old corticospinal pathways^6^. It is possible that the new and old corticospinal-network systems might be preserved across species^49^.

Our results provided a biological model that the organization of the corticospinal motor networks was related to behavioral laterality of hand-use preference. Morphological studies in post-mortem brains showed that the subregion of left M1 that send the corticospinal motor fibers has larger volumes than that of the right M1 in right-handed humans^50–52^. When the intrinsic hand muscles are targeted with TMS, motor excitability is lower in the left M1 than in the right M1^53^. Together, the left M1 likely has more developed direct corticospinal connections than does the right M1. Furthermore, the left M1 is more excitable in people with a higher degree of right-handedness^53^. Our results supported the model that the direct corticospinal projections from the left M1 are the main network constituents during RHM (Supplementary Fig. 9). Moreover, a greater weight on the contralateral direct corticospinal network was correlated with the preference to right-hand use (Fig. 4). No previous studies addressed whether the asymmetric involvement of the indirect corticospinal network related to the lateralization of hand preference. The network analysis favored the model that LHM recruited the indirect corticospinal network more conspicuously than did RHM (Fig. 3) although LHM also recruited the direct corticospinal network. Furthermore, the interindividual variance of the recruitment of the indirect corticospinal network was correlated with the lateralization of hand preference. Our results posit an idea that the asymmetric recruitment of the indirect corticospinal network might underlie the lateralization of the hand use, especially through the control of non-preferred hand. We thus proposed the model representing the asymmetric organization of the direct and indirect corticospinal networks linked with the lateralization of hand preference. Previous studies suggested that the laterality of hand preference has been observed at the infancy or childhood^2,41,54^. Since we did not study the development of the direct and indirect networks, it remains to be elucidated if the asymmetric recruitment of the direct and indirect corticospinal network results from the prenatal factor^44^, the postnatal use-dependent factor^55^, or both.

Previous studies suggested the contralateral M1 and the ipsilateral M1 played different roles in controlling hand motor tasks^13–15^. Activation of the contralateral M1 was consistent across various motor tasks. However, activation of the ipsilateral M1 in movement varies with tasks, depending upon the requisite level of fine control, force, and distal/proximal muscles^14,47,48^. The present task showed a slight deactivation in the ipsilateral M1 (Supplementary Fig. 3) while other tasks increased activity in the ipsilateral M1^46,47^. For instance, a pervious report revealed that activation in the ipsilateral M1 induced reduction of SHc activity^32^. These results suggested that deactivation and activation in the ipsilateral M1 may differently influence spinal cord activity. Future studies should investigate how the involvement of the indirect corticospinal network might differ in different motor tasks.

Using the corticospinal fMRI techniques, we were able to computationally test the feasibility of the indirect corticospinal pathways that corresponded to the transsynaptic integration of the signals from both M1s. Note, however, that we were not able to directly demonstrate the anatomical or neurophysiological substrates of the monosynaptic or multi-synaptic corticospinal pathways reported from studies in non-human primates^5,56^. The present corticospinal fMRI techniques do not have a temporal resolution fine enough to examine different latencies in transsynaptic transmission of the signals in the direct and indirect networks^57^. The present corticospinal fMRI does not have a spatial resolution good enough to identify activity from the relay neurons located in the brainstem or the spinal cord^58^. At this moment, we thus have acknowledged the technical limitations of the corticospinal fMRI techniques and it remains elusive whether our network models of the direct and indirect corticospinal pathways might share the substrates with the monosynaptic or multi-synaptic corticospinal pathways observed in non-human primates. Further refinement of the corticospinal fMRI techniques with a higher temporal or spatial resolution will be needed to overcome this issue, and the in-depth investigation of the direct and indirect corticospinal networks in humans is of interest at the next research step.

In conclusion, this corticospinal fMRI study presented here has suggested the network model representing the asymmetric organization of the corticospinal networks for unilateral hand movement that underlie the lateralization of hand preference. We speculate that the developmental asymmetry of SHc maturation may trigger the lateralization of the corticospinal motor system, which may influence the asymmetric organization of the cerebral hemispheres^8,9^. This study opens a new avenue of research to explore how the lateralized corticospinal motor system which underlies the unique human trait of handedness may have triggered the acquisition of additional evolutionary gains in human beings over other primates.

## Methods

### Participants and study design

We recruited 16 right-handed, healthy young adults with mean age of 21.4 years (range 18–26 years; 7 males, 9 females). None of the participants reported any history of neuropsychiatric disorders. Three participants were excluded after we found severe artifacts in their imaging data (see “Preprocessing”). The data from the remaining 13 participants (5 males, 8 females) were analyzed and reported. The 13 participants were judged as right handed using the EHI^13^, by which the mean laterality quotient and standard deviation was 84.0 ± 13.4 (range 53.8–100). The participants were informed about the experimental procedure, and all of them participated in the experiment after giving written informed consent according to the study protocol approved by the National Center of Neurology and Psychiatry ethics committee.

In this experiment, the participants were required to gaze at the fixation point on a monitor, and to perform a repetition of tapping with the thumb and small finger of either the right or left hand after the presentation of an auditory cue every second (i.e., 1 Hz; Presentation software, Neurobehavioral Systems, Albany, CA, USA). The task took 11 s, and alternated between RHM and LHM. The task blocks were interleaved with resting blocks of 28.6 s each, in which participants were instructed not to move the fingers of either hand, although auditory cues were paced at the same frequency (i.e., 1 Hz) as during the task. In total, the entire experiment consisted of 8 blocks for the task and 9 blocks of rest. Before beginning the experiment, participants practiced the finger tapping outside the magnetic resonance imaging (MRI) scanner to ensure that were familiar enough with the task to perform it well in the scanner.

In the MRI scanner, the participants’ arms were aligned with the sides of their body and their hands were placed in a supine position. The bilateral upper limbs and the bodies of the participants were fixed to the scanner bed with band restraints to minimize joint and neck movements during the finger-tapping task. Participants were also instructed to relax and remain still in order to minimize motion artifacts and imaging noise at the level of the cervical spinal cord.

To ensure that the participants performed the task using the assigned hand without mirror movements with the opposite hand, electoromyography (EMG) signals were visually observed in real time from the bilateral muscles of the deltoid, the brachialis and brachioradialis, the abductor policis brevis, and the abductor digiti minimi and recorded using BrainAmp ExG MR (Brain Products, Gilching, Germany). Surface electrodes with shielded plates and cables were placed over the muscles with an interelectrode distance of approximately 2 cm, and a ground electrode was placed on the dorsal surface of the right wrist.

### Data acquisition

We used a 3 Tesla MAGNETOM Verio MRI scanner (Siemens, Erlangen, Germany) with standard 12 channel head coil and 4 channel neck array coil to measure signal changes in regions of interest including M1 in both hemispheres and the cervical spinal cord. For simultaneous scanning of the two distant areas in one single volume, we applied acquisition of multiple slices along a sagittal plane covering from top of the head to the upper thoracic spinal cord at the segmental level of Th1 along a rostro–caudal axis. We acquired the BOLD sensitive, gradient-echo, echo planar imaging (EPI) sequence combined with Generalized Autocalibrating Partially Parallel Acquisition. EPI parameters were as follows: repetition time = 2600 ms; echo time = 25 ms; flip angle = 75 degrees; acceleration factor for GRAPPA = 2; rectangular field of view, 190 (anterior–posterior) × 320 (rostral–caudal) mm; matrix size = in-plane resolution of 2.5 (anterior–posterior) × 2.5 (rostral–caudal) mm^2^; slice thickness = 3 mm (left–right) and 44 slices. Supplementary Fig. 1 shows an example of the functional image acquired with the present imaging protocol.

### Functional MRI data analysis

#### Preprocessing

We collected data from 16 participants but excluded three participants for the following reasons: severe N/2 ghosting (*n* = 2) and severe distortion artifact in the spinal cord (*n* = 1). Thus, the results were obtained from the remaining 13 participants (5 males, 8 females). The functional MRI (fMRI) data were preprocessed using the free distribution software SPM8 (http://www.fil.ion.ucl.ac.uk/spm/) and FSL (https://fsl.fmrib.ox.ac.uk/fsl/fslwiki/FSL). The first eight volumes of fMRI run were discarded to allow the MRI signal to reach T1 equilibrium. Preprocessing steps included motion correction and slice timing correction to the first slice.

Correction for residual motion was performed for all functional images using the SPM standard six parameter rigid body transformation. As compared with the brain fMRI data, spinal fMRI is much more susceptible to artifacts derived from the cerebrospinal fluid (CSF) and other biological factors. This increased susceptibility in spinal fMRI can be explained by the small cross-sectional cord area where fMRI signals can be contaminated by faint motion and by pulsation of surrounding CSF; magnetic field inhomogeneity causing distortion and ghosting artifacts in the encoding direction; and physiological noises due to respiration and cardiac beats masking BOLD signals in the spinal cord^54–57^. Therefore, to detect BOLD signals in the spinal cord, it is ideal to remove as much physiological noise as possible^54–56^. To do this, we used the independent component analysis in FSL (Multivariate Exploratory Linear Optimized Decomposition into Independent Components) for decomposing the components corresponding to noise and artifacts^59,60^. Through this procedure, we removed biological noise and corrected several artifacts related to motion and CSF pulsation^61^.

Most fMRI studies have focused on brain activity and the required spatial normalization of an individual’s brain images to the anatomical standard template to localize regional activity for analysis of group data. For the brain imaging data, fMRI images were spatially normalized to fit the Montreal Neurological Institute template based on the standard stereotaxic coordinate system to obtain the group level coordinates of M1 and other areas. All images were smoothed with an isotropic Gaussian kernel of 4 mm full width at half maximum. Low-frequency noise was removed with high-pass filter, and serial correlations were adjusted using an autoregressive model.

However, there is no consensus yet for the methodology to spatially normalize individual images from spinal cord scans, although previous studies have attempted to develop a methodology^5,6,57^. Therefore, we decided not to apply spatial normalization, and instead estimated spinal activity on the individual’s anatomical space. It is widely accepted from surgical exploration that the segment of the spinal cord at C7 and Th1—the region of interest activated by the finger-tapping task—is located at the same level of the vertebral bodies of C6 and C7^62^. Thus, for determining its anatomical localization, we referred to the spinal segments as the anatomical landmarks.

#### Voxel-wise analysis

We performed voxel-wise analysis to search for activated voxels in bilateral M1 and SHc. Vectors representing the experimental paradigm were modeled for the individual-level general linear model analysis through convolution of a boxcar function (task duration: 28.8 s) with the canonical hemodynamic response function. Six parameters (3 translations and 3 rotations) representing the motion of the head and spinal cord were included in the design matrix as covariates of no interest. Then we tested main effects of hand movement (i.e., RHM or LHM relative to the rest period) in each participant.

Activation in the spinal cord was examined in individual-level analysis (see “Preprocessing”). The threshold was set at uncorrected *p* = 0.05 using a one sample *t* test [17]. The volume of interest (VOI) for SHc was defined as the activated voxels in the anatomical template for each participant (see VOI analysis). Activation in bilateral M1was tested in group level analysis after spatial normalization of the individual participants’ fMRI data. The individual-level contrast images were obtained from each participant and then used for the group-level analysis. The group analysis was done using a one sample *t* test. The family wise error (FWE) corrected threshold was set at *p* = 0.05 with use of the small volume correction (SVC) method^19^.

#### VOI analysis

To investigate task-evoked activation in the spinal cord, we computed beta estimate values from the individual level analysis and extracted individuals’ BOLD time course data within activated voxels in bilateral M1 and SHc. The VOIs of the spinal segments C7-Th1 and C5 were drawn manually by hand in each participant by referring to the individual’s coregistered anatomical T1 image. The VOIs were separated in the midline between the right and left hemiside of the spinal cord that probably included both the gray and white matter. The β values were averaged among voxels within the VOIs for RHM and LHM, and then averaged across participants. Three-way repeated measures ANOVA with factors “hand” (RHM/LHM), “segment” (C5/C7-Th1) and “side” (right/left) on β values were examined (see Fig. 1) for stronger activity in the C7-Th1 compared with the C5 segment (i.e., “segment” effect), and also for stronger activity in the hemisphere ipsilateral to the moving hand compared with the contralateral hemisphere (i.e., “side” effect). Posthoc comparisons were also done using the Holm–Bonferroni method^63^. The time series data was used for effective connectivity and functional connectivity analyses (see Fig. 1, Supplementary Figs. 4–6 and supplementary note 1). To improve the signal-to-noise ratio in the spinal activity data, a dataset of the mean of 11 time points was computed by averaging each time point across four blocks of each hand movement in each individual.

To estimate bilateral M1 activity, the beta estimate values and time series data for individuals were computed in their VOIs in the same way as described above. The only difference was that the VOIs were created on the standard anatomical template. The group level, voxel-wise analysis was performed. We searched for the peak coordinate of M1 activity inside Brodmann area 4 based on the anatomical atlas (http://fmri.wfubmc.edu/software/pickatlas), with a FWE corrected threshold of *p* < 0.05. The three dimensional, sphere shaped VOIs were created with an 8 mm radius centered at its peak coordinate for the right or left M1 in each participant. The estimate beta values were examined for replication of bilateral M1 activity during RHM or LHM (Supplementary Fig. 3). The dataset of the mean of 11 time points with respect to bilateral M1 was computed in the same way as described above.

#### Effective connectivity analysis and functional connectivity analysis

Effective connectivity and functional connectivity analyses were performed using a paired dataset of the mean of 11 time points with respect to activity in bilateral M1 and SHc. Because variance differed between regions, time series data for an individual participant was transformed to z scores in each region for both connectivity analyses. Note that raw, nontransformed data of SHc activity and M1 activity is depicted in Fig. 1, Supplementary Fig. 2, and Supplementary Fig. 3 for display purposes. In the effective connectivity analysis, single linear regression analysis was performed to compute the value of R^2^ and the regression slope in each participant using the time series data in M1 and SHc during hand movement and rest. We identified the task-dependent change in effective connectivity by comparing regression slopes between hand movement and rest within participants. In the functional connectivity analysis, correlation analysis was performed using the same time series dataset in the two regions during RHM or LHM and rest in each participant. To estimate presence of the functional connections relevant to the task, the correlation coefficient (*r*) was compared between hand movement and rest within participants. The within subject comparison for the functional and effective connectivity analyses was made using the Wilcoxon signed-rank test.

### Network model analysis

Regression analyses were used to examine how interaction of the influences derived from contralateral and ipsilateral M1 might determine the net influences between bilateral M1 and the spinal cord. We hypothesized two forms of interactions. First, as in previous studies^22^, we examined how interaction of activity in bilateral M1 might affect activity in SHc ipsilateral to the moving hand (see Supplementary Fig. 7 and refer to the equation corresponding to the interaction model). Next, the interaction of connectivity terms was tested to examine how integration of connectivity of the contralateral M1-SHc and ipsilateral M1-SHc networks might determine net connectivity between bilateral M1 and SHc (see Supplementary Fig. 8 and refer to the equations corresponding to the interaction models). Since the number of terms differed between models, the adjusted *R*^2^ was computed. The bootstrap procedure was used to statistically compare models. The null distributions were created by the following simulation methods using the original dataset: choosing arbitrary participants, swapping the data with respect to the activity and slope separately within the chosen participants and then computing the value of *R*^2^, and the slope on multiple regression analysis based on the simulated data. Significance was set to values beyond 95% confidence intervals estimated from the simulated null distributions.

The simulation analysis was applied to the selected network model for RHM and LHM in order to estimate an individual’s differences in the involvement of the direct and indirect corticospinal networks (Fig. 4 and supplementary note 2). We predicted that the involvement of the direct and indirect corticospinal networks might be associated with preferred right hand and non-preferred left hand, respectively. The weight for each term was optimized so that residual errors were minimized for each individual in the model. The constraints for the weights were set on the basis of the results acquired from the group level network model analysis (Fig. 1 and Supplementary Fig. 8): the weights for the contralateral and ipsilateral direct corticospinal network model were positive (i.e., more than zero). The weight was negative for the indirect corticospinal network model (i.e., less than zero). Values of the weights for each individual are shown in Fig. 4. We investigated the correlation between the weights of the direct or indirect corticospinal networks and the degree of hand preference. Hand preference was estimated by the EHI questionnaire. The values of their weights and the EHI scores were used in the correlation analysis.

We further sought to examine the relationship between functional connectivity/effective connectivity and the degree of hand preference (Supplementary Note 3), although our results did not support interhemispheric functional interactions between M1s during RHM or LHM in the employed task (Supplementary Note 1). We performed the simple correlation analysis with use of the values of the functional connectivity/effective connectivity (Supplementary Note 1) and the EHI scores.

### Correlation analysis

We used correlation analyses to compute the correlation coefficient (*r*) and its statistical significance (Fig. 4, Supplementary Figs. 3–6, and Supplementary Notes 1, 3; details for each analysis are in the main text, figure legends, and supplementary data). The M1 activity and SHc activity were transformed to z-scores with respect to each variable and then tested. The significance threshold was set at *p* < 0.05.

### Statistics and reproducibility

All analyses used data from all 13 participants. A two-tailed test was used in all of the analyses: either the Wilcoxon signed-rank test or Pearson’s correlation coefficient (Figs. 1, 4, Supplementary Figs. 3–6, Supplementary Fig. 9, and Supplementary Notes 1, 3). The highest and lowest 95% confidence interval was determined from the simulated null distributions. F values, t values, and the degree of freedom for all results are provided in the figures.

Analyses in the present study were performed using the SPSS software package (IBM, Chicago, IL, USA), and the MATLAB Statistics and Machine Learning Toolbox 8.1 and Optimization Toolbox 8.1 (Release 2017b, The MathWorks, Inc., Natick, MA, USA).

### Reporting summary

Further information on research design is available in the Nature Research Reporting Summary linked to this article.

## Supplementary information

Supplementary Information

Description of Additional Supplementary Files

Supplementary Data 1

Reporting Summary

## Data Availability

The following pieces of data from individual participants are available online (https://drive.google.com/drive/u/0/folders/1dWuVpZ5ogrY3EVbUdgjeg5aqwNis3QFQ): the EHI index (Fig. 4), BOLD activity in bilateral M1 and SHc (Fig. 1 and Supplementary Fig. 3), the regression slope obtained from regression analyses (i.e., effective connectivity; Figs. 1, 4, Supplementary Figs. 6, 8, and Supplementary Notes 1, 2 and 3), and the correlation coefficient computed from correlation analyses (i.e., functional connectivity; Supplementary Figs. 3–6 and Supplementary Note 1). These data were the basis for the network analyses. The weights calculated in the network analyses (Fig. 4, Supplementary Fig. 9 and Supplementary Note 2) are also provided online.

## Code availability

We employed the corrcoef, regress, and fmincon MATLAB functions for the regression analyses, correlation analyses, and network analyses. The codes implementing the functions are provided from the corresponding authors upon request.
