# Supplementary material for: A computational model based on corticospinal functional MRI revealed asymmetrically organized motor corticospinal networks in humans
Source: Commun Biol. 2022 Jul 5;5:664. doi: 10.1038/s42003-022-03615-2 (PMC9256686; doi:10.1038/s42003-022-03615-2)
Supplement: Supplementary file 1 — Supplementary Information [file 42003_2022_3615_MOESM1_ESM.pdf]

Supplementary information

Title: A computational model based on corticospinal functional MRI revealed asymmetrically organized motor corticospinal networks in humans

Authors: Eiji Takasawa [1,2]†, Mitsunari Abe [1]*†, Hirotaka Chikuda [2], Takashi Hanakawa [3,1]*

Affiliations:

[1] Department of Advanced Neuroimaging, Integrative Brain Imaging Center, National Center of Neurology and Psychiatry, Tokyo, Japan

[2] Department of Orthopaedic Surgery, Gunma University Graduate School of Medicine, Maebashi, Gunma, Japan.

[3] Department of Integrated Neuroanatomy & Neuroimaging, Kyoto University Graduate School of Medicine, Kyoto, Japan

*Correspondence to: Mitsunari Abe (mitsunari.abe.a8@ncnp.go.jp) or Takashi Hanakawa (hanakawa.takashi.2s@kyoto-u.ac.jp)

Supplementary Figure 1. Echo planar imaging acquired with multiple slices along a sagittal plane. This protocol allowed the scanning of regions of interest in one single volume including

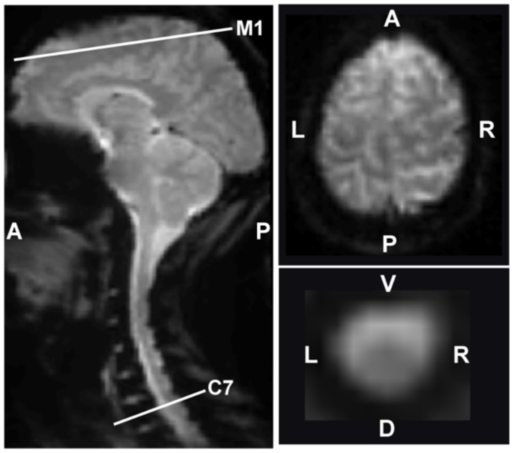

M1 in bilateral hemispheres and the cervical spinal cord. The left panel shows the sagittal planes along the longitudinal body axis (i.e. rostro–caudal) covering the brain hemisphere and the spinal cord. A = anterior side. P = posterior side. Right panels show axial images of bilateral M1s (see the while line at the level of hand area in M1, left panel) and the spinal cord (see while line at the segmental level of C7, left panel) that were reconstructed from the raw data consisting of the sagittal slices. L = left side. R = right side. Upper and lower side of the axial images correspond to the anterior and posterior side of the brain or the ventral and dorsal side of the spinal cord. V = ventral side. D = dorsal side. For display purposes, the axial view of the reconstructed images has been magnified by 3.

Supplementary Figure 2. Segment-specific and side-specific activity in the spinal cord during hand movement. Spinal activation maps at the segments of C7–Th1 from a representative participant (left panels, uncorrected $p < 0.05$ for display purposes) during RHM (upper panel) and LHM (lower panel). L = left side. R = right side. Bar graphs (right panels) represent the mean and standard error of the mean of beta estimate values (arbitrary unit, a.u.) averaged across participants in contralateral or ipsilateral SHc at the segmental level of C5 or C7-Th1 with respect to the moving hand. * $p < 0.05$. n.s. = not significant. contra-SHc = contralateral SHc. ipsi-SHc = ipsilateral SHc.

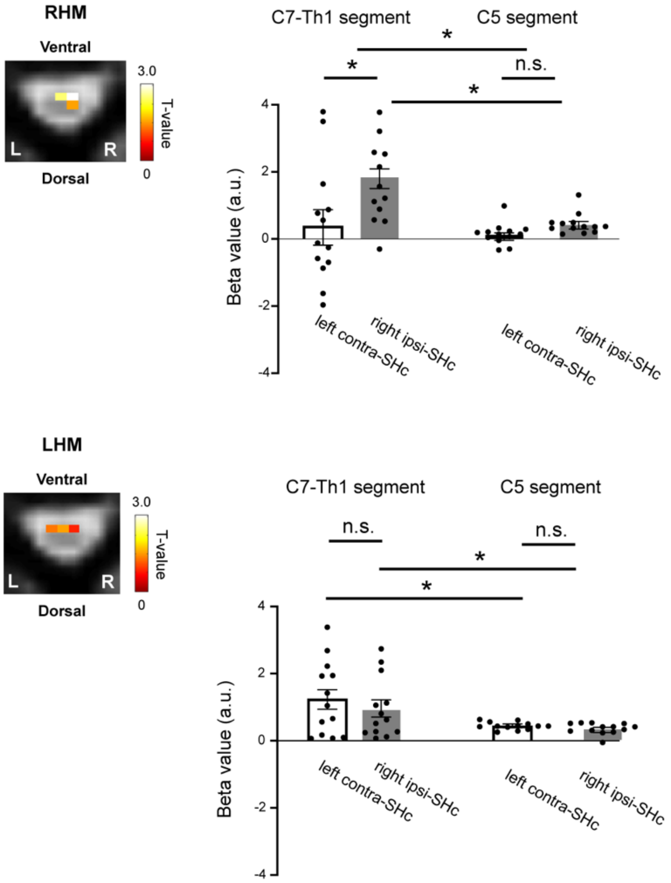

ANOVA table

Interaction	
SGEMNET * SIDE * HAND	F(1,12) = 1.88, p = 0.196
SGEMNET * HAND	F(1,12) = 0.099, p = 0.76
SGEMNET * SIDE	F(1,12) = 0.49, p = 0.50
**SIDE * HAND**	**F(1,12) = 5.71, p = 0.034**
main effect	
**SGEMNET**	**F(1,12) = 6.72, p = 0.024**
HAND	F(1,12) = 0.03, p = 0.87
SIDE	F(1,12) = 2.97, p = 0.110

The effects of SEGMENT $_{(C5/C7–Th1)}$, SIDE $_{(contralateral/ipsilateral)}$ and HAND $_{(right\ hand/left\ hand)}$ on SHc activity were computed using three way ANOVA. The main effect of SEGMENT (i.e., C7–Th1 activity was greater than C5 activity; $F_{(1, 12)} = 6.72$; $p = 0.024$) and the interaction effect of SIDE * HAND ($F_{(1, 12)} = 5.71$; $p = 0.034$) were significant. See also the results for the other interaction terms in the ANOVA table. In a confirmatory volume of interest (VOI) analysis, right and left SHc at the C5 segment did not

show increased activity during either RHM or LHM ($p > 0.4$ for all VOIs). During RHM, the C7–Th1 segment showed higher activity in right ipsilateral SHc ($p = 0.013$), but not in left contralateral SHc ($p = 0.44$), than did the C5 segment. During RHM, The C7–Th1 segment showed greater activity in ipsilateral SHc than in contralateral SHc ($p = 0.008$). During LHM, the C7–Th1 segment showed higher activity than the C5 segment in both contralateral SHc ($p = 0.020$) and ipsilateral SHc ($p = 0.040$). Comparable activity was observed between ipsilateral SHc and contralateral SHc ($p = 0.72$). Thus, both RHM and LHM induced the segment-specific contralateral SHc activity.

Supplementary Figure 3. Activity in primary motor cortex (M1) contralateral and ipsilateral to the moving hand. Voxel-wise, whole-brain analysis was used to investigate activity in M1 in both hemispheres. RHM and LHM activated contralateral M1 as compared to rest. For left M1 during RHM, peak MNI coordinates (x, y, z) = -34, -20, 58, T = 7.94, family-wise error (FWE) corrected p = 0.03. For right M1 during LHM, peak MNI coordinates (x, y, z) = 38, -16, 52, T = 7.85, FWE corrected p = 0.03. To estimate ipsilateral M1 activity, analysis was performed in the spherical VOIs with 8 mm radius centered at the peak coordinate of left M1 or right M1. RHM suppressed activity in right ipsilateral M1 relative to rest (T = 5.35, FWE corrected p = 0.04), while LHM did not induce suppression of left ipsilateral M1 activity (uncorrected p > 0.1).

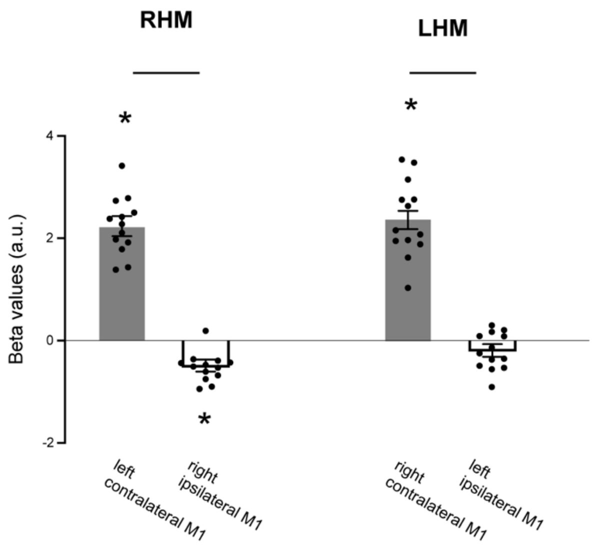

VOI analysis was used to examine different activities between the contralateral and ipsilateral M1. The beta estimate values were computed within the 8 mm sphere centered at the peak coordinate of left or right M1. The bar graphs showed the mean and standard error of the mean of beta estimate values (arbitrary unit, a.u.) averaged across participants in contralateral or ipsilateral M1 with respect to the moving hand. * p < 0.05. contra-M1 = contralateral M1. ipsi-SHc = ipsilateral M1. Each dot indicated the individual's data. Activity was higher in contralateral M1 than in ipsilateral M1 during either RHM or LHM (paired t-test of left M1 vs. right M1 during RHM, p = 0.0002; paired t-test of left M1 vs. right M1 during LHM, p = 0.0002). Reduction of activity in ipsilateral M1 during RHM was greater than during LHM

(paired t-test of left M1 vs. right M1, $t_{12} = -2.54$, $p = 0.026$). Note individual's data in ipsilateral M1 during RHM or LHM. During RHM, the ipsilateral M1 showed deactivated (i.e. below the zero) in all of the participants except one. During LHM, it showed deactivated in 9 out of the 13 all participants while 4 participants showed active. We thus interpreted a trend of the deactivation of the ipsilateral M1 during LHM, although the size of the group-level effect was smaller during LHM than during RHM.

Supplementary Figure 4. Correlation coefficient in the contralateral M1−SHc network during hand movement relative to rest. Bar graphs represent the mean and standard error of

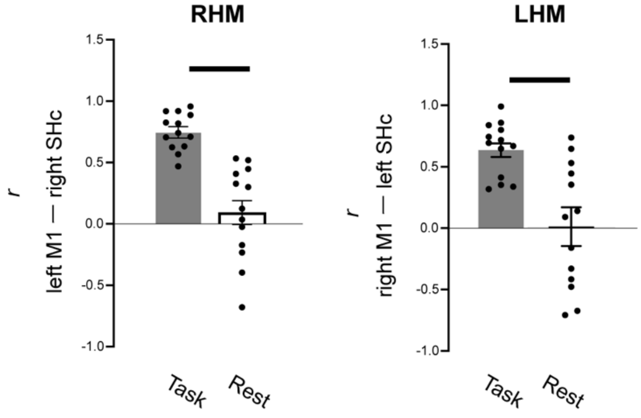

the mean of the correlation coefficient (*r*) between contralateral M1 activity and ipsilateral SHc activity during hand movement or rest (RHM, $r = 0.75 \pm 0.04$; rest, $r = 0.09 \pm 0.11$. LHM, $r = 0.64 \pm 0.06$; rest, $r = 0.014 \pm 0.09$). Correlation coefficient (*r*) was higher during hand movement relative to rest, regardless of the moving hand (RHM vs. rest, $p = 0.033$. LHM vs. rest, $p = 0.043$).

Supplementary Figure 5. Correlation coefficient in the ipsilateral M1−SHc network during hand movement relative to rest. Bar graphs represent the mean and standard error of the mean

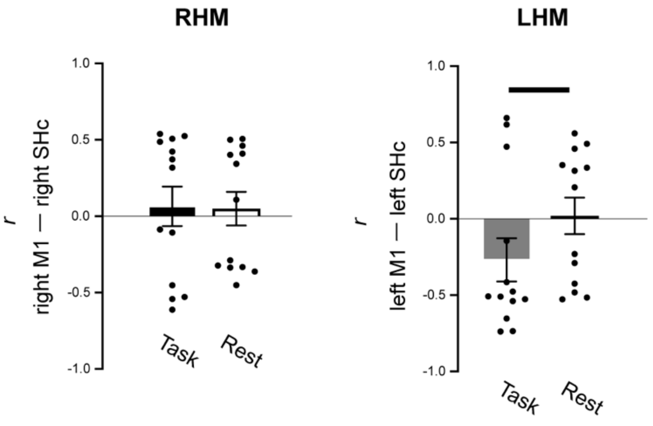

of the correlation c oefficient (*r*) between ipsilateral M1 activity and ipsilateral SHc activity during hand movement or rest (RHM, movement, $r = 0.065 \pm 0.13$; rest, $r = 0.049 \pm 0.11$. LHM, movement, $r = -0.29 \pm 0.09$; rest, $r = 0.06 \pm 0.10$). LHM increased the correlation coefficient (*r*) in an inverse manner during LHM (movement vs. rest, $p = 0.010$), whereas RHM did not affect the coefficient (movement vs. rest, $p = 0.78$).

Supplementary Figure 6. Contralateral M1 activity and effective connectivity of the ipsilateral network between M1 and SHc. Relationship between contralateral M1 activity (x axis) and effective connectivity of the ipsilateral M1–ipsilateral SHc network (y axis), in which each dot indicates a single participant's data. (a) The higher the right contralateral M1 activity, the greater the negative value of the effective connectivity of the ipsilateral M1–ipsilateral SHc network during LHM (right panel, correlation coefficient, *r* = -0.58, p = 0.03; simple linear regression analysis, regression slope = -0.58; 95% confidence intervals -0.57 to 0.13). (b) Such a relationship was not evident between contralateral M1 activity and effective connectivity of the ipsilateral M1–ipsilateral SHc network during RHM (left panel, p = 0.39).

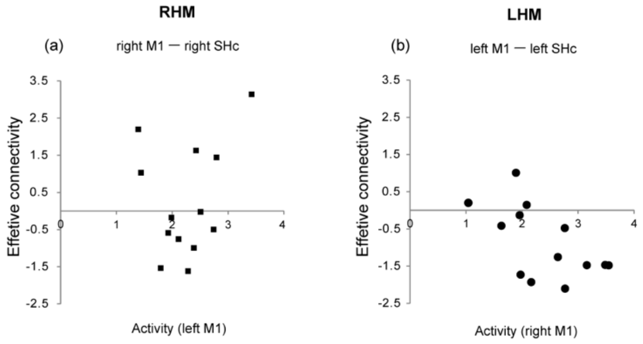

Supplementary Note 1. Functional connectivity and effective connectivity between bilateral M1s.

Simple correlation analysis was performed between bilateral M1 activity during hand movement or rest. The mean and standard error of the mean of the correlation coefficient (r) are as follows: RHM, movement, r = -0.07 ± 0.17; rest, r = 0.027 ± 0.11. LHM, movement, r = -0.095 ± 0.12; rest, r = 0.013 ± 0.08. Task induced modulation of interhemispheric functional connectivity was not found during either hand movement (Wilcoxon signed-rank test, RHM, p = 0.55; LHM, p = 0.57).

Simple linear regression analysis was computed between bilateral M1 activity during hand movement or rest. The mean and standard error of the mean of the regression slope are as follows: RHM, slope = -0.13 ± 0.11; rest, slope = 0.03 ± 0.08. LHM, slope = -0.04 ± 0.26; rest, slope = 0.03 ± 0.14). Neither RHM nor LHM modulated effective connectivity between bilateral M1 (Wilcoxon signed-rank test, RHM, p = 0.38; LHM, p = 0.50).

Supplementary Figure 7. Models of activity in ipsilateral SHc during LHM.

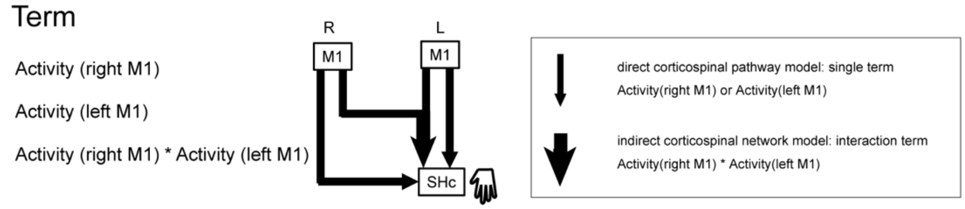

Activity(right M1)	Activity(left M1)	Activity(right M1) * Activity(left M1)	*Adjusted R^2*	*F*	*p value*
+	+	+	- 0.18	0.38	0.77
–	+	+	- 0.07	0.62	0.55
+	–	+	- 0.12	0.38	0.70
+	+	–	- 0.15	0.24	0.79
–	–	+	- 0.02	0.76	0.40
–	+	–	- 0.05	0.40	0.54
+	–	–	- 0.07	0.26	0.62

We examined whether full models with interaction of activity between bilateral M1s—activity (contralateral M1) * activity (ipsilateral M1)—or simpler models consisting of activity (contralateral M1) or activity (ipsilateral M1) either explained ipsilateral SHc activity during left hand movement. See the component with respect to each term in the network model. R = right hemisphere. L = left hemisphere. Multiple regression analysis showed that neither type of model was significant ($p > 0.4$). Information regarding AIC or BIC was not computed in this analysis because it was evident that no models explained SHc activity. The "+" indicates that the corresponding term was included in the models, the "–" indicates that the term was not included. Adjusted R^2, F values, and p values are reported.

Supplementary Figure 8. Network model analysis of connectivity between bilateral M1 and ipsilateral SHc during LHM.

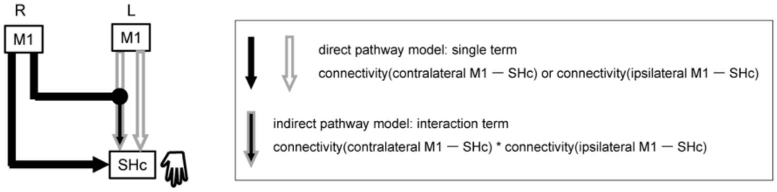

		Ipsilateral direct network	Contralateral direct network	indirect network (ipsilateral M1 + contralateral M1)	Adjusted R^2	F	p value	AIC	BIC
δ	a	+	+	+	0.477	4.56	0.055	40.76	43.56
	b	+	+	-	0.05	1.32	0.31	47.95	49.64

δ $\Delta R^2 = 0.427$ (95 % confidence intervals, lower threshold = - 0.28, higher threshold = 0.34)

We tested whether the interactions between connectivity of contralateral M1–ipsilateral SHc and connectivity of ipsilateral M1–ipsilateral SHc may mediate modulation of activity between SHc and bilateral M1. Previous studies suggested that bilateral M1s played different roles in controlling of hand movements [4-6]. We modelled the separate influences of the contralateral M1 and the ipsilateral M1 on SHc activity in the indirect corticospinal network. The overall influences of the indirect corticospinal network from bilateral M1s onto the spinal cord represented the combination of the influences from the contralateral M1 and those from the ipsilateral M1. The equation for models that contained all terms with interactions is as follows: activity(SHc) = α * connectivity(contralateral M1– ipsilateral SHc) * activity(contralateral M1) + β * connectivity(ipsilateral M1– ipsilateral SHc) * activity(ipsilateral M1) + γ * ({interaction [connectivity(contralateral M1– ipsilateral SHc) * connectivity(ipsilateral M1– ipsilateral SHc)] * activity(contralateral M1)} + interaction {[connectivity(contralateral M1– ipsilateral SHc) * connectivity(ipsilateral M1– ipsilateral SHc)] * activity(ipsilateral M1)}) + ε, where α, β and γ are weights for explanatory variables and ε indicates residual errors. Provided previous observations in a human study report [32], we assumed comparable influences from the contralateral M1 or the ipsilateral M1 onto the spinal cord through the indirect corticospinal

network. We thus assigned the same weight for the two components of the interaction term multiplied by the contralateral M1 or the ipsilateral M1 activity. We performed the group-level estimate of the likely network models including the interaction terms that may explain SHc activity (see also Fig. 3). The models were constructed so that the same weights were assigned to all participants for each of the interaction terms and the single terms. The table shows a list of the two model equations tested here, labelled "a" or "b", with included terms indicated with "+". Adjusted R^2, F values, and p values are listed for all of the models. AIC and BIC were also computed to estimate the most likely model. The models with and without the interaction terms were compared (paired comparisons are labelled "δ"), and the differences in adjusted R^2 (ΔR^2) are reported. The comparison between models was considered to be statistically significant if R^2 was beyond the threshold at the 95% confidence intervals computed from simulations using bootstrap procedures.

Supplementary Note 2. Simulation analysis of SHc activity during hand movement.

The simulation analysis was performed to estimate the individual participants' differences in the involvement of the direct and indirect corticospinal networks. Our results indicated that LHM involved not only the contralateral and ipsilateral corticospinal networks (Fig. 1) but also the indirect corticospinal network (Fig. 3). Hence, those three components were all included in the model here. We used the same model for both of RHM and LHM. The equation is as follows: activity (SHc) = α * connectivity(contralateral M1–ipsilateral SHc) * activity(contralateral M1) + β * connectivity(ipsilateral M1– ipsilateral SHc) * activity(ipsilateral M1) + γ * (interaction {[connectivity(contralateral M1– ipsilateral SHc) * connectivity(ipsilateral M1– ipsilateral SHc)] * activity(contralateral M1)} + interaction {[connectivity(contralateral M1– ipsilateral SHc) * connectivity(ipsilateral M1– ipsilateral SHc)] * activity(ipsilateral M1)} + ε. The weights for each term were determined so that the residual errors were minimized for each individual in the model.

Supplementary Figure 9. Simulation analysis of recruitment of the contralateral direct corticospinal network during hand movement. Our previous analyses (see Fig. 3 and supplementary figure 6) supported the involvement of the contralateral direct corticospinal network during movement of either hand. We examined if the component of the contralateral direct corticospinal network may explain SHc activity to a greater extent during RHM than during LHM. The weight computed in supplementary figure 6 indicated the degree to which each network component may predict SHc activity. The weights for the contralateral direct corticospinal network were compared between RHM and LHM by Wilcoxon signed-rank test, and were found to be higher during RHM than during LHM ($p = 0.0021$).

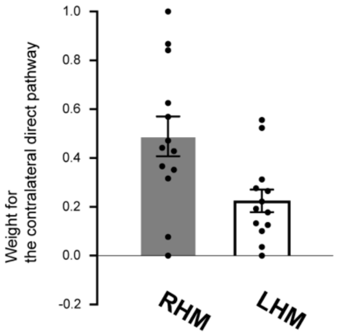

Supplementary Note 3. Functional connectivity and effective connectivity in either hemisphere of M1 or between bilateral M1. We did not find evidence supporting interhemispheric functional connections between M1s during RHM or LHM in the employed task (supplementary note 1). We further sought evidence that participant variability in interhemispheric influences between bilateral M1 might not be associated with the differences in the degree of hand preference between individuals. First, we examined the relationship between preference for the right hand and functions of the interhemispheric M1 projection. Next, we tested the relationship between nonpreference for the left hand and functions of the interhemispheric M1 projection. Both the regression slope and the correlation coefficient (r), estimated through the individual-level analysis, were used as measures of effective connectivity and functional connectivity, respectively. There was no evidence that the EHI score was correlated with interhemispheric effective connectivity or with functional connectivity between bilateral M1 during RHM or LHM ($p > 0.3$ for all analyses). Finally, we investigated whether bilateral M1 activity or interaction between M1 in both hemispheres might be correlated with the degree of hand preference, but these parameters were not correlated with hand preference ($p > 0.2$ for all analyses).
