# Supplementary material for: A computational model based on corticospinal functional MRI revealed asymmetrically organized motor corticospinal networks in humans
Source: Commun Biol. 2022 Jul 5;5:664. doi: 10.1038/s42003-022-03615-2 (PMC9256686; doi:10.1038/s42003-022-03615-2)
Supplement: Supplementary file 4 — Reporting Summary [file 42003_2022_3615_MOESM4_ESM.pdf]

nature research

Corresponding author(s): Mitsunari Abe

Last updated by author(s): May 7, 2022

Reporting Summary

Nature Research wishes to improve the reproducibility of the work that we publish. This form provides structure for consistency and transparency in reporting. For further information on Nature Research policies, see Authors & Referees and the Editorial Policy Checklist.

Statistics

For all statistical analyses, confirm that the following items are present in the figure legend, table legend, main text, or Methods section.

n/a	Confirmed	
☐	☒	The exact sample size (n) for each experimental group/condition, given as a discrete number and unit of measurement
☐	☒	A statement on whether measurements were taken from distinct samples or whether the same sample was measured repeatedly
☐	☒	The statistical test(s) used AND whether they are one- or two-sided<br>*Only common tests should be described solely by name; describe more complex techniques in the Methods section.*
☒	☐	A description of all covariates tested
☐	☒	A description of any assumptions or corrections, such as tests of normality and adjustment for multiple comparisons
☐	☒	A full description of the statistical parameters including central tendency (e.g. means) or other basic estimates (e.g. regression coefficient) AND variation (e.g. standard deviation) or associated estimates of uncertainty (e.g. confidence intervals)
☐	☒	For null hypothesis testing, the test statistic (e.g. F, t, r) with confidence intervals, effect sizes, degrees of freedom and P value noted<br>*Give P values as exact values whenever suitable.*
☒	☐	For Bayesian analysis, information on the choice of priors and Markov chain Monte Carlo settings
☐	☒	For hierarchical and complex designs, identification of the appropriate level for tests and full reporting of outcomes
☐	☒	Estimates of effect sizes (e.g. Cohen's d, Pearson's r), indicating how they were calculated

Our web collection on statistics for biologists contains articles on many of the points above.

Software and code

Policy information about availability of computer code

Data collection	3 Tesla MAGNETOM Verio MRI scanner (Siemens, Erlangen, Germany) ; BrainAmp ExG MR (Brain Products, Gilching, Germany); Presentation ver16.3 (Neurobehavioral Systems, Albany, CA, USA)
Data analysis	MATLAB Statistics and Machine Learning Toolbox 8.1 and Optimization Toolbox 8.1 (Release 2017b, The MathWorks, Inc., Natick, MA, USA); SPSS software package version 22.0 (IBM, Chicago, IL, USA)

For manuscripts utilizing custom algorithms or software that are central to the research but not yet described in published literature, software must be made available to editors/reviewers. We strongly encourage code deposition in a community repository (e.g. GitHub). See the Nature Research guidelines for submitting code & software for further information.

Data

Policy information about availability of data

All manuscripts must include a data availability statement. This statement should provide the following information, where applicable:

- Accession codes, unique identifiers, or web links for publicly available datasets
- A list of figures that have associated raw data
- A description of any restrictions on data availability

The following pieces of data from individual participants are available online (https://drive.google.com/drive/u/0/folders/1dWuVpZ5ogrY3EVbUdgjeg5aqwNis3QFQ): the EHI index (Fig. 7), BOLD activity in bilateral M1 and SHc (Fig. 1, Fig. 2, and S-5), the regression slope obtained from regression analyses (i.e., effective connectivity; Fig. 3), and the correlation coefficient computed from correlation analyses (i.e., functional connectivity; S-2 to S-4). These data were the basis for the network analyses. The weights calculated in the network analyses (Fig. 6, Fig. 7, and S-5 to S-7) are also provided online.

Field-specific reporting

Please select the one below that is the best fit for your research. If you are not sure, read the appropriate sections before making your selection.

☐ Life sciences ☒ Behavioural & social sciences ☐ Ecological, evolutionary & environmental sciences

For a reference copy of the document with all sections, see nature.com/documents/nr-reporting-summary-flat.pdf

Behavioural & social sciences study design

All studies must disclose on these points even when the disclosure is negative.

Study description	functional magnetic imaging study that measured activity in cerebral cortex and the spinal cord during right-hand or left-hand movement. We examined asymmetry in recruitment of the functional connectivity between primary motor cortex and the spinal cord during right-hand or left-hand movement
Research sample	We recruited undergraduate and graduate students from several universities in Tokyo, JAPAN. They were both males and females with a mean age of 21.3. None of the participants reported any history of neuropsychiatric disorders. All of the participants were judged as right handed using the Edinburgh Handedness Inventory.
Sampling strategy	We chose convenience sampling for recruiting participants. We performed calculation to estimate the sample size for conducting t-test and simple regression analysis.
Data collection	The Presentation software (Neurobehavioral Systems, Albany, CA, USA) controlled the timing of presentation of auditory stimuli and the timing of instruction of each hand condition (i.e. right-hand or left-hand condition). We used 3 Tesla MAGNETOM Verio MRI scanner (Siemens, Erlangen, Germany) to measure activity in the cortex and the spinal cord. We also employed electromyography (BrainAmp ExG MR, Brain Products, Gilching, Germany) to observe muscle activity during hand movement or rest.
Timing	Between June 1, 2012 and September 30, 2013
Data exclusions	Three participants were excluded after we found severe artefacts in their imaging data.
Non-participation	No participants dropped out.
Randomization	Participants were not allocated into experimental groups.

Reporting for specific materials, systems and methods

We require information from authors about some types of materials, experimental systems and methods used in many studies. Here, indicate whether each material, system or method listed is relevant to your study. If you are not sure if a list item applies to your research, read the appropriate section before selecting a response.

Materials & experimental systems

n/a	Involved in the study	
☒	☐	Antibodies
☒	☐	Eukaryotic cell lines
☒	☐	Palaeontology
☒	☐	Animals and other organisms
☒	☐	Human research participants
☒	☐	Clinical data

Methods

n/a	Involved in the study	
☒	☐	ChIP-seq
☒	☐	Flow cytometry
☐	☒	MRI-based neuroimaging

Magnetic resonance imaging

Experimental design

Design type	Block design
Design specifications	Total of 4 blocks for each of right-hand (RHM) or left-hand movement (LHM) condition in each subject. T). The task block took 11 s, and alternated between RHM and LHM. The task blocks were interleaved with resting blocks of 28.6 s each. In total, the entire experiment consisted of 8 blocks for the task and 9 blocks of rest.
Behavioral performance measures	No measurements. Electoromyography (EMG) signals were visually observed in real time from hand muscles to ensure that that the participants performed the task using the assigned hand without mirror movements with the opposite hand.

Acquisition

Imaging type(s)	functional
Field strength	3 Tesla
Sequence & imaging parameters	gradient-echo, echo planar imaging (EPI) sequence combined with Generalized Autocalibrating Partially Parallel Acquisition. EPI parameters were as follows: repetition time = 2600 ms; echo time = 25 ms; flip angle = 75 degrees; acceleration factor for GRAPPA = 2; rectangular field of view, 190 (anterior–posterior) × 320 (rostral–caudal) mm; matrix size = in-plane resolution of 2.5 (anterior–posterior) × 2.5 (rostral–caudal) mm2; slice thickness = 3 mm (left–right) and 44 slices.
Area of acquisition	For simultaneous scanning of the two distant areas (primary motor cortex and the spinal cord9 in one single volume, we applied acquisition of multiple slices along a sagittal plane covering from top of the head to the upper thoracic spinal cord at the segmental level of Th1 along a rostro–caudal axis

Diffusion MRI ☐ Used ☒ Not used

Preprocessing

Preprocessing software	The functional MRI (fMRI) data were preprocessed using the free distribution software SPM8 (http://www.fil.ion.ucl.ac.uk/spm/) and FSL ver5.0.8 (https://fsl.fmrib.ox.ac.uk/fsl/fslwiki/FSL). Default parameters were used for slicetiming correction, realignment and corregistration. Images were smoothed with an isotropic Gaussian kernel of 4 mm full width at half maximum.
Normalization	Linear normalization was performed for brain imaging data. Normalization was NOT performed for the spinal cord data.
Normalization template	Brain images were spatially normalized to fit the Montreal Neurological Institute template.
Noise and artifact removal	We used the independent component analysis in FSL (Multivariate Exploratory Linear Optimized Decomposition into Independent Components) for decomposing the components corresponding to noise and artefacts.
Volume censoring	We employed the fsl_glm command whereby MRI signals was corrected by removing non-biological signals that were explained by head motions, or were likely derived from white matter or cerebral spinal fluid.

Statistical modeling & inference

Model type and settings	Voxel-based fMRI analysis in the whole brain: random effect model in the second level
Effect(s) tested	We employed a three way ANOVA and post hoc analysis to test side-specific and segment-specific activity in the spinal cord during hand movement.

Specify type of analysis: ☐ Whole brain ☐ ROI-based ☒ Both

Anatomical location(s): hand motor area in the primary motor cortex was determined on the basis of the anatomical atlas (http://fmri.wfubmc.edu/software/pickatlas)

Statistic type for inference (See Eklund et al. 2016)	voxel wise report
Correction	Posthoc comparisons were also done using the Holm–Bonferroni method

Models & analysis

n/a	Involved in the study
☐	☒ Functional and/or effective connectivity
☒	☐ Graph analysis
☐	☒ Multivariate modeling or predictive analysis

Functional and/or effective connectivity	In the effective connectivity analysis, single linear regression analysis was performed to compute the value of R2 and the regression slope in each participant using the time series data in primary motor cortex M1 and the spinal cord during hand movement and rest.<br>In the functional connectivity analysis, correlation analysis was performed using the same time series dataset in the two regions during right-hand or left-hand movement and rest in each participant.
Multivariate modeling and predictive analysis	We performed structural equation modeling analysis to test if interaction of influences from primary motor cortices in bilateral hemispheres might explain activity in the spinal cord. Independent variables were activity in primary motor cortex, and effective connectivity between primary motor cortex and the spinal cord. Dependent variable was activity in the spinal cord.
